# Barriers to cardiovascular disease secondary prevention care in the West Bank, Palestine – a health professional perspective

**DOI:** 10.1186/s13031-018-0165-x

**Published:** 2018-07-16

**Authors:** Jane Collier, Hanna Kienzler

**Affiliations:** 1 Cardiovascular Rehabilitation Team, Guy’s & St. Thomas’s Hospital NHS Foundation Trust, 1st Floor Becket House, 1 Lambeth Palace Road, London, SE1 7EU UK; 2 Department of Global Health & Social Medicine, Room 2.7a East Wing, School of Global Affairs Faculty of Social Science and Public Policy, King’s College London, Strand, London, WC2R 2LS UK

**Keywords:** Non-communicable diseases, Cardiovascular disease, Social determinants of health, Secondary healthcare, Risk factors, Behaviour change, War, Palestine

## Abstract

**Background:**

Non-communicable diseases (NCDs) - including cardiovascular disease (CVD), cancer and diabetes - have become a significant global burden on health. Particularly concerning are CVD rates, causing approximately 18 million deaths worldwide every year. The statistics show that the disease is no longer a predominantly high-income country phenomenon, but affects, increasingly, countries in both developing regions and conflict-affected areas. In the occupied Palestinian territory (oPt), the focus of this article, CVD ranks top of ten NCD killers, accounting for approximately 37.6% of deaths. Key risk factors discerned in *primary* care settings have been related to both structural determinants (i.e. the Israeli occupation) and individual behavioural factors. Unfortunately, no data are available for *secondary* care settings in the region and, consequently, little is known about patients and their capacity for risk factor behaviour change to manage their CVD.

To begin closing this gap in knowledge, our study provides insight into cardiovascular disease secondary prevention care with the overall aim to enhance the understanding of the complexities of managing NCDs like CVD in conflict-affected settings. Specifically, research was carried out among Palestinian health professionals who specialise in coronary artery disease in the West Bank to elicit their views on (a) how socio-political, health system and individual behavioural factors might hinder patients to change their health behaviour and impact on the provision of healthcare and (b) possible solutions for overcoming identified barriers to behaviour change on societal as well as individual-patient levels within secondary care provision in a context of protracted conflict.

**Methods:**

This study is based on a qualitative approach in order to provide more in-depth information about health beliefs and behaviours, experiences and views of health professionals with regards to CVD secondary care. In total, 12 semi-structured interviews were conducted among doctors providing treatment to patients with CVD in secondary care settings. Interviews focused on health professionals’ perspectives on risk factors and perceived barriers to behaviour change among known CVD patients receiving secondary care. Interviewees were also asked to propose possible actions that could be taken to overcome the identified barriers at both societal and individual patient levels. All interviews were digitally recorded, transcribed and analysed using thematic analysis.

**Results:**

Study results confirmed our prior theory of the complex entanglement of socio-political, health system and individual-level factors with regards to CVD experience, health-seeking and treatment. Also confirmed was our assumption that it is crucial to understand experts’ definitions and approaches to treatment in order to grasp their visions for appropriate and improved prevention and treatment options. In particular, study participants highlighted how *political determinants*, notably the detrimental impact of the Israeli occupation, and *social determinants*, directly and indirectly influence *behavioural determinants* due to physical and bureaucratic barriers to accessing health facilities, economic hardship and chronic stress. These stressors, in turn, were perceived as having a negative effect on individual behavioural risk factors including smoking, unhealthy diet and an increasingly sedentary lifestyle. Proposed solutions included more focused interventions from the Ministry of Health as well as surveillance, primary prevention and health promotion, and management to positively effect behaviour change in order to address the growing burden of CVD in the region.

**Conclusions:**

The study has highlighted medical professionals’ perceptions of how structural and individual behavioural determinants influence their own and individual patient’s abilities to manage cardiovascular risk factors in a setting affected by chronic conflict. Consequently, we propose that medical and social intervention strategies generally used to address CVD risk, be strategically adapted in order to be useful and effective in contexts of armed conflict. Specifically, we call for a solid understanding of the socio-political context and existing health services as well as health providers’ and patients’ health beliefs and related behaviours when developing future health options aimed at addressing CVD in the region. Moreover, for health provision to be effective as well as sustainable, attention needs to be given above all towards a solution for political change.

## Background

Non-communicable diseases (NCDs) have become a significant global burden on health due to the rise in rates of conditions such as cardiovascular disease (CVD), cancer and diabetes. NCDs now form one of the major causes of mortality and morbidity globally with 38 million deaths worldwide [1]. Within this context, CVD is a key contributor to the rise, causing approximately 18 million deaths worldwide every year [2]. While previously the increase in incidence and mortality rates have affected mostly high-income countries, a new trend is emerging as NCDs and particularly CVD are now being experienced by regions previously considered low-risk for such diseases, namely low- and middle-income countries (LMICs) and conflict-affected areas, particularly in the Middle East and North African region (MENA) [3–6]. Within the MENA countries, NCD mortality rates vary, with Lebanon experiencing an 85% NCD mortality rate (of which 47% are CVD-related) and Jordan experiencing a 76% NCD mortality rate (of which 35% are CVD-related) [7]. In this region, too, high levels of behavioural risk factors have been discerned such as daily tobacco smoking (26.3% in Jordan, 18.9% in Egypt, 37.6% in Lebanon, and 19.3% in Palestine); insufficient physical activity (46.8% in Lebanon and 46.5% in Palestine); and obesity (34.3% in Jordan, 28.2% in Lebanon, 34.6% in Egypt, and 26.8% in Palestine) [5].

In the occupied Palestinian territory (oPt), the focus of this article, research has found that heart disease, cerebrovascular disease and cancer are currently the main causes of morbidity and mortality [8]. Consequently, there are high direct costs of care, high indirect costs in loss of production and much societal stress [8]. In terms of provision of *primary* healthcare in the West Bank, research has found that the most dominant feature impacting on access to healthcare is the Israeli occupation and, related to this, the separation wall between Israel and the West Bank, the myriad numbers of Israeli military checkpoints as well as invasions, detentions and land confiscation [9–14]. Additionally, the underfunding and suboptimal organisation of the existing healthcare system have been connected to a lack of both skilled health professionals and affordable essential medicines which make the provision of preventive and curative health provision for NCDs challenging [15–17]. This inadequate healthcare response has been linked to rising health inequities and social injustice on a large scale as poorer communities are less well served by the health system despite having higher rates of CVD risk factors [8].

Disconcertingly, little, if any, research has been undertaken in the field of *secondary* healthcare within the oPt, despite the fact that current reports highlight that this should be urgently tackled [18]. Insight into secondary healthcare is crucial as it has been shown to contribute to an improved multi-sectoral strategy that has the capacity to positively impact on health outcomes and to reduce the associated economic burden of cardiovascular disease [19]. To begin closing this gap in knowledge, our study focuses on cardiovascular disease secondary prevention care with the overall aim to enhance the understanding of the complexities of managing NCDs, like CVD, in conflict-affected settings. Specifically, research was carried out among health professionals who specialise in coronary artery disease (angina and heart attack) in the West Bank to elicit their views on (a) how socio-political, health system and individual behavioural factors might hinder patients to change their health behaviour and impact on the provision of healthcare and (b) possible solutions for overcoming identified barriers to behaviour change on societal as well as individual patient levels within secondary care provision in a context of protracted conflict. In the following, relevant background information will be provided on NCDs in the context of armed conflict and, specifically, in the oPt. Thereupon, empirical findings will be presented with the aim of providing new insights into barriers, policies and available services related to cardiovascular disease in the West Bank. The discussion and conclusion will contribute to and expand on earlier findings on the rise in NCDs, notably CVD, within the oPt and highlight how political, social and behavioural determinants are perceived to impact on people’s health and wellbeing. Underlying this is an argument for the urgent need for robust data-gathering systems in conjunction with multi-sectoral approaches to the delivery of healthcare in the region, notwithstanding the cultural and socio-political implications.

### NCDs in the context of armed conflict

Armed conflicts around the world are increasingly affecting civilian populations [20], and changes in warfare technology have led to an upsurge in injuries, diseases and psychological traumas among affected populations [21]. At the same time, conflict-affected communities are experiencing increasing urbanisation and ageing populations, with older persons physically less able than most other adults to ensure their health and wellbeing [22–24]. These changing demographics have led to shifts in the disease burden with a noticeable increase in NCD prevalence rates [25–27].

Individuals experiencing chronic disease conditions and their psychosocial consequences are particularly at risk when exposed to armed conflict and insecurity related to family disintegration, environmental degradation, dislocation of food production systems and disruption of the local economies [28–32]. Research has convincingly shown that these direct and indirect factors lead to a decrease in the quality of healthcare and increase in distress [30, 33]. For example, Clark et al. [34] link the chronic stress of political conflict to diseases like diabetes and heart problems. This study supports the notion of chronic health consequences resulting from the stress of living under political conflict and adds to the discussion regarding the effects of war on civilians [35, 36].

Despite these insights, the humanitarian response to emergencies has traditionally focused on the management of acute conditions such as infectious illnesses and physical and psychological trauma and the provision of relatively short-term emergency relief and aid, rather than long-term care [37]. NCDs, on the other hand, have largely been neglected by humanitarian aid providers [38], and the provision of long-term care has been particularly challenging in emergency settings as conflicts decrease the ability of health systems to respond [27]. Reports have shown that such limited access to timely treatment can lead to poor outcomes for patients, as NCDs need to be managed continually in order to achieve best outcomes. In the context of the Middle East (Afghanistan, Libya, Syria and Yemen), the Safeguarding Health in Conflict Coalition [39] has highlighted the negative impact of war and violence on health systems. They found that hospitals were closed or even destroyed; the provision of healthcare was irregular; and staff and supplies were largely lacking. Consequently, the population was considered to be at increased risk with regards to mortality and morbidity due to chronic diseases such as diabetes, asthma, kidney disease and cardiovascular disease. Based on their findings, the Coalition calls for approaches to preventive and curative health responses in conflict settings to evolve substantially within primary, secondary and tertiary care.

### NCD experience in the context of the occupied Palestinian territory

In the occupied Palestinian territory, health is powerfully influenced by political, economic and social determinants [9, 11]. Particularly NCDs have been seen as a key challenge for the Palestinian health system, and the burden has been shown to be increasing over the past decade [40–43]. Additional research on incidence, prevalence and increasing burden of risk factors for NCDs in Palestine has identified adverse developments for the most common conditions including hypertension [44–47], diabetes [48–51] and obesity [52–59], and these are now noted to be common conditions among the local population. With particular reference to CVD, the Palestinian Ministry of Health (MoH) reports that the disease ranked top of ten NCD killers, accounting for 29.5% of deaths in 2014 [60].

Drivers of this increase in NCDs are reported as primarily connected to both structural determinants, including the Israeli occupation, and individual behavioural factors such as increased smoking rates (particularly amongst young women), lack of opportunity for physical activity, and obesity. In another report, the Palestinian MoH [61] highlighted poverty, unemployment and transitions in food consumption patterns as contributing to the increasing prevalence of these behavioural risk factors, all of which are related to the development of NCDs, especially CVD. A study by Sousa and Hagopian [12] notes that the Israeli checkpoints and road blocks, separation wall and military presence in Palestine limit access to medical equipment and medicine, compromise the education of healthcare professionals and block access to both preventive and curative health services. They argue that political and socio-economic instability are the main barriers to the success of the MOH’s healthcare agenda as these factors are leading to an inability to provide appropriate follow-up healthcare to manage NCDs. Additionally, the study highlights that “(Palestinian) patients often could not afford medication due to increased unemployment and poverty resulting from the expropriation of land, destruction of businesses and homes, and loss of free movement for work” (p. 525). Such deficiencies are reported to exacerbate the conditions of patients suffering from chronic diseases such as diabetes or hypertension - the biggest risk factors for cardiovascular disease. Similarly, a report by the Safeguarding Health in Conflict Coalition [39] highlights the Israeli occupation as an important barrier to health provision stating, “Israeli security forces created new checkpoints and refused to allow priority of passage to Palestinian ambulances until receiving authorization through bureaucratic channels, thereby delaying the transport of patients who were in the midst of emergencies by up to an hour” (p. 10)*.* Additionally, Palestinian hospitals are reported to be raided regularly (to make arrests or to collect medical files and patient information) disturbing patient care and intervention.

It has been well documented that Palestinian patients require permits to travel to areas where many of them may be referred for further medical investigation, specialised treatment, surgery and other interventions. In addition to the permit requirement – which may take some time to receive and in many cases is refused – Palestinians are restricted in their mode and point of entry at the 98 fixed checkpoints in the West Bank (as of 31st January 2017) inhibiting their freedom of movement and right to health still further [10, 12, 13]. According to a WHO Special Report [14], the Israeli Civil Administration (ICA) denied health access, or delayed responding to requests, to one in five Palestinian patients seeking referral health care in 2011 and 2012, citing ‘security’ as the reason for denial.

To address some of the problems outlined above, the Palestinian MoH had previously developed a health plan with a particular focus on CVD, obesity-related diabetes and cancer. Unfortunately, its implementation was unsuccessful partly due to a lack of coordination between the various stakeholders involved working for the ministry and various non-governmental organisations [52]. More recently, the MoH, with support from WHO, has developed a national policy and strategy to prevent and manage NCDs based on a recognised need for both primary and secondary prevention [62]. Whilst covering legislation, regulation and inter-sectoral policy objectives, this plan does not specifically identify secondary care plans. This shortcoming might be partly related to the lack of locally relevant research evidence on risk factors, barriers and treatment outcomes in secondary care settings.

## Methods

This study employed a qualitative research design as an appropriate methodology to inquire into structural and individual barriers to achieving lifestyle modifications recommended for CVD secondary prevention among patients, from the perspective of Palestinian medical professionals based in secondary care. Particular attention was paid to health professionals’ beliefs, experiences and views with regards to CVD secondary care and the ways in which they connected these to broader contextual factors including the political, social and economic determinants of health [63, 64]. The study was carried out over a period of five weeks in 2015.

Study participants included 12 doctors aged between 26 and 63 who were predominantly male except for two female doctors. They held main appointments with private (5) and governmental healthcare services (5) as well as health NGOs (2) located in Ramallah (7), Nablus (1), Bethlehem (2), and East Jerusalem (2). Younger study participants identified as ‘residents’ and their length of qualification as a doctor ranged from two to four years; all of them worked in governmental hospitals. Doctors working in the private sector, on the other hand, had been qualified for between ten and 38 years and were more experienced in the field of cardiac investigations, diagnostics and subsequent interventions compared to their younger colleagues in the government health sector. The two doctors working in NGOs had both been qualified for over 12 years. All study participants provided cardiac interventions (i.e. coronary angiography), surgical interventions (i.e. stenting and coronary artery bypass grafting) as well as follow-up appointments to their patients.

Sampling for this study followed a purposeful and snowball sampling approach. Specifically, invitation emails were sent to ten main medical facilities providing cardiac interventions in the West Bank. Individual responses were received from three that were consequently followed up with further details of the study and information sheets. Staff from these institutions then connected the first author with colleagues working for other health facilities in government, private and NGO sectors.

Semi-structured interviews were conducted by the first author using a topic guide focusing on risk factors, barriers to healthcare and possible solutions for improved healthcare. Interviews were conducted in English except for one that was conducted in Arabic with a Palestinian translator. The interviews lasted between 40 and 70 min and were, with the consent of study participants, audio recorded and later transcribed verbatim by the first author. The data from the transcripts was subsequently coded inductively in order to understand the basic social processes arising within the study, and to begin to build categories and themes from the codes. Specifically, the coded data were, first, categorised and linked by relationship. Thereupon, links were established between the categories and properties defined such as phenomena, causal conditions, context, action strategies and consequences. Through an interpretative process, it was possible to identify the core categories that structure the following results section.

### Study limitations

This study aimed to include between eight and 15 health professionals currently working in the field of cardiovascular disease, particularly in secondary prevention care. It was hoped to obtain a selection of different health professionals to include doctors, nursing staff, physiotherapists, occupational therapists, psychologists and other relevant healthcare workers. However, it became apparent that this type of healthcare within the West Bank is only carried out by medical doctors, hence all interviewees being limited to that profession. Furthermore, it was intended for the sample to consist of an equal number of female/male participants. Unfortunately, gatekeepers mainly selected male colleagues and, therefore, only two out of the 12 interviews were conducted with female respondents. While it was not possible to establish the exact ratio of female to male doctors in Palestine, it is anticipated that there are likely to be far fewer female doctors than male, simply as a result of the historical culture of women in Arab societies and the worldwide domination of males within the medical profession. Nevertheless, it would have been important to interview more female doctors to provide, if any, differences in their perspectives of providing a service within a male-dominated profession and how this may impact on their ability to address the subject of secondary care. Different responses may have been given by female respondents to questions about differences between male and female patient behaviour, reflecting upon the lower status of women in that society. The language of respondents is also important in considering understanding, transparency and validity. All 12 interviews apart from one were conducted in English. Palestinian doctors’ training is carried out in English therefore there is a shared understanding of medical terminology. There were, however, occasions when Arabic words were used to indicate something particular within the Palestinian culture in order to emphasise certain practices. The terms were later translated with the help of a Palestinian translator. The one interview carried out with a translator did provide the translator with an opportunity to contribute his own views and some personal answers to the interview questions, but these were not used as part of the transcription process or as valid evidence.

## Results

Health providers painted a fairly bleak picture of secondary care provision. As will become apparent, they related the current situation mainly to structural (political, social, economic and health systems) and individual behavioural risk factors, which deterred their patients from participating in lifestyle changes that could improve health outcomes from cardiovascular events. At the same time, the practitioners made concrete recommendations for actions that could be taken to overcome some of the less macro-political barriers on the system and individual patient levels, and to address problems identified within health provision itself.

### Structural risk factors and barriers to healthcare

All study participants referred to stress and insecurity as the main risk factors for the notable increase of CVD in the West Bank. According to them, stress was related to (a) structural determinants, particularly to the Israeli occupation, with its political instability, lack of freedom of movement and military presence and (b) daily stressors including economic hardship, unemployment and family conflicts. A doctor said, for instance:

I don’t think it’s just one thing, sometimes you’d get the stress from, you know, directly occupation-related, and sometimes you get the stress from the social economical status which is indirectly related to the occupation.

Stress and insecurity were also related to feelings of hopelessness for the future and depression. One doctor from a private practice indicated that 20% of his patients were prescribed antidepressants while another one working for a large governmental facility noted:

Living under occupation means not much to look forward to, occupation is (a) chronic disease.

Stress was considered to be associated particularly with the separation wall and Israeli checkpoints, both of which often prevent patients from reaching appropriately specialised health facilities in time. A doctor from a governmental hospital outlined the problem for patients who need urgent medical treatment as follows:

If someone has (a) heart attack who comes to (the) hospital you hope that they present on time. The ideal of the recommended ‘door to open’ time of 90 min, or the ambulance to emergency room time of 1 h is laughable here. The patient needs a permit, basically a visa to visit [the] hospital. How could somebody with a heart attack get a visa? And if he comes, the physician needs a permit to reach the hospital. So it’s a ‘no win’ situation.

Besides these political determinants, low socioeconomic status was considered an important factor impacting on self-care and, related to this, health outcomes. On the one hand, low salaries meant that patients lacked the capacity to purchase medication and, on the other, their long working hours did not permit them to engage in physical activity. One study participant explained:

The working hours are ridiculous and then, you know, you don’t have enough time to go and do something (...) you don’t have the time to go to the gym.

Even if patients were to consider the possibility of addressing such lifestyle changes, there was a reported lack of public spaces for recreation and leisure, and the local environment was described as deficient of suitable facilities for physical activity. Someone explained:

In Bethlehem, walking here, it’s dusty, there’s lots of cars, it’s uphill [laughing]. There’re no parks for people to walk around.

Also doctors in other West Bank cities lamented a lack of recreational areas and facilities that are designed for the use of adults, adolescents and children.

### Individual behavioural risk factors for CVD

Study participants referred to a number of behavioural risk factors for CVD including smoking, a sedentary lifestyle and diet and explained how they were exacerbated by the protracted political conflict and related stress. Smoking was recognised as a major issue that should be tackled to reduce the risk of cardiovascular disease:

Smoking is really a big, big problem here (…), maybe it could amount to about 70% of adult men who smoke in this part of the world.

Whilst all respondents considered a reduction in smoking important, they also highlighted that it was extremely difficult for patients to change this behaviour as they used smoking as a means to combat the stress of living within a conflict zone and as a form of enjoyment. One respondent suggested that, due to the fact that people’s circumstances are challenging, one of their escapes and possible means of enjoyment is through smoking.

Because living under occupation is the main issue here, you know, people are experiencing a hard life so cigarettes are an escape.

It was further noted that especially older patients were reluctant to quit smoking because they considered it to be a way of socialising and bringing some form of enjoyment into their occupation-affected and often insecure lives. Younger smokers, on the other hand, were believed to smoke mainly because they followed family patterns of smoking behaviour, and once started, it was a difficult behaviour to stop. In addition, several participants alluded to the worrying trend of the increasing number of women smoking shisha pipes, known as *argila.*

Another contributing factor to cardiovascular disease was felt to be the increasingly sedentary lifestyle of many Palestinian people. A particularly low uptake of physical exercise was noted due to the fact that it does not play an important role in people’s everyday lives. “Exercise?” one of the doctors laughed, “Zero, we don’t do it here really”. This participant suggested that exercise is not an activity that is carried out within the Palestinian society habitually and on a wide scale. Numerous respondents reflected that the sedentary lifestyle contributes to the rise in obesity. It was noted to be of particular prevalence among women patients. For instance, two doctors highlighted that one of the reasons why there were more women presenting with CVD was because their lifestyle was believed to be more sedentary than that of men, that is, they were less active because they stayed at home more. This could imply that these health providers viewed women in a stereotypical female role and that being responsible for the home and childcare are viewed as ‘sedentary’ activities. It was also suggested that there might be an increased presentation of CVD amongst women because they tend to stay at home when ill and are believed to visit hospitals more rarely. This could result in delayed hospital presentations by which time some risk factor complications may have already set in.

Furthermore, a change in dietary habits among patients was believed to lead to diabetes, obesity and high cholesterol. This was often attributed to the influence and transference over time to a Westernised diet such as high-fat fast foods and sugary drinks particularly by younger people who now opt for this in preference to traditional home-cooking. A health provider reasoned:

Now, unfortunately we’re getting slowly into this (…) Westernised lifestyle. This means obesity and all its problems such as diabetes, high cholesterol and a sedentary lifestyle. These all lead to cardiac diseases being the number one problem here.

The cultural change in the lifestyle habits of patients was identified especially by the older study respondents who, over their long years of working in cardiovascular medicine, were seeing more women smoking and noticing less healthy dietary choices. Correspondingly, many of the younger doctors also seemed to be aware that these cultural changes presented a significant problem for tackling risk factors, but felt that the patients’ lack of motivation was a key factor in encouraging lifestyle change.

A number of doctors stated that, despite apparent patient awareness of the benefits of adopting healthier lifestyles, this lack of motivation was compounded by a level of complacency around family illness history relating to CVD. It was felt this near denial led to many patients not considering making lifestyle changes until after the occurrence of their own heart attack. Doctors also felt this complacency accounted for the noticeable increase in younger patients now presenting with CVD as, despite having a positive family history, young people were not addressing any required behavioural changes to reduce their own risk of CVD. This noted self-regard in the young was connected to a lack of motivation, in part due to people’s circumstances:

It’s the lack of motivation which (…) I would say under the current circumstances, (…) could not be changed. It is more difficult because when you look at the circumstances (…), the political conditions in general, there is not much hope for the younger generation .

This poor motivation for behaviour change, often driven by low mood, was believed to exist despite apparent patient awareness of the benefits of adopting healthier lifestyles.

### Proposed solutions for improved secondary care provision

Respondents reflected upon what kinds of changes would be necessary and feasible to help people change their behaviour under these challenging circumstances. In particular, they highlighted the importance of developing multi-disciplinary teams in their clinics, improving communication and referral structures, increasing human and material resources, engaging more in advocacy and, importantly, policy development..

Health providers explained that they would be able to work more effectively if they could work in multi-disciplinary teams with specialists from various relevant departments. One practitioner commented that this would help him to:

Take my decision in the next step (of patient) management, this would be better for the patient. I feel there is a shortage of physiotherapists, dieticians and nurses, and this affects my work actually.

It was also suggested that working more closely with specialists from other health departments would improve the handover and continuation of patient treatment. Several doctors commented on the particular need for endocrinologists to address the increasing presentation of diabetic patients with complications, as this could affect the onset of CVD.

This lack of integrated healthcare was also connected to the importance of improving communication between and within health departments. One practitioner said for instance:

We have a defect here in our system in that not every patient is followed by the same specialist and so I guess there’s a defect in the communication between the doctors here in our department.

This poor communication was also reported between primary and secondary healthcare providers. A doctor outlined:

Actually there is no satisfaction, there is no good co-operations between the hospital and the diabetic centre for primary healthcare, it is above our ER but we don’t have any communication.

In terms of a possible solution to this problem the same doctor explained:

We should include it together, the endocrinologist in the diabetes centre (…) and with cardiologist for acute coronary syndrome to manage the risk factor from there and we manage the acute events.

Other respondents echoed the importance of working across specialisations as it was felt to be crucial for monitoring, follow-up and on-going education for lifestyle support such as smoking cessation and dietary adherence.

In order to further improve secondary healthcare for CVD, several health practitioners emphasised the importance of having enough staff on hand. Yet it is important to note that they differed in their views on whether or not there were enough medical experts available to assess and care for patients with CVD. Half of them considered there to be enough residents in their clinic while others highlighted a lack of doctors and specialists, such as those working in endocrinology, who could do more to provide support for diabetic patients. It was noticeably the younger practitioners working in the governmental hospitals who complained about a lack of specialists.

In addition to a lack of staff, respondents referred to a shortage of equipment. This, it was explained, led to many patients with known risk factors for cardiovascular disease not being supported in monitoring their condition appropriately, and not receiving adequate advice that may prevent complications or the leading to chronic disease. As a result, respondents stated that there is a higher presentation of patients to secondary care facilities once complications have set in and a rise in the presentation of younger patients with cardiovascular disease. In addition, doctors felt that patients were confused about where to go for treatment and often bypassed initial services and, instead, headed straight to secondary care. It was believed that another reason they may present initially at secondary care was due to patient concerns about the current state of primary care services. Consequently, patients preferred to attend secondary care facilities as they had more ‘trust’ in the hospital service.

Besides the faults highlighted within the primary care provision leading patients to default to secondary care services, doctors suggested other solutions to support patients to manage their CVD such as advocacy, research and health policy. In terms of advocacy, they argued that regardless of patient reluctance to behaviour change, a doctor’s most important role was education and reaching a compromise with patients about lifestyle change and treatment options. It was considered that education could be provided through the doctor-patient relationship or via social media. One study respondent stated that he carried out regular radio presentations where he focused on particular risk factors for CVD and provided advice for listeners. However, some respondents noted the relevance of the educational level of patients to be able to understand and follow advice:

The good news is our society is one of the most-educated societies in the whole Arab world (…), but at the same time there are areas in Palestine where people are much less educated and this means the compliance (...) and you talk to the family or somebody who’s well educated in the family you might (still) get problems in their understanding of the disease, and the need of follow up.

Reference was made by participants to the importance of evidence to inform priority setting. Two doctors noted a lack of published statistics to support the widely acknowledged increase in the presence of CVD risk factors:

We don’t have national statistics, at least good ones, to be sure what we are seeing.

This could be significant as potentially, without the knowledge of the number of patients presenting with risk factors, it may be difficult to consider how to plan, prioritise and fund health conditions.

Relating to an identified possible solution at policy level by participants, it was suggested that more could be done particularly within the food and tobacco industries. In relation to tobacco, one doctor commented:

It’s difficult because you need to go another ten, twenty years to be able to enforce that there is no smoking in public places.

This would imply that any potentially helpful policy changes take a long time to implement.

With regard to the food industry, mention was made of there being two types of restaurants – a costly one offering low calorie food, and a cheaper one with high calorie content. This was felt to be unfair as busy, hardworking or impoverished people would visit the cheaper restaurants and, thereby, subject themselves to a diet that was not considered to be in the least bit helpful in reducing the risk of CVD.

## Discussion

Focusing on the views of Palestinian health professionals on barriers and resources for CVD secondary care prevention in the West Bank, this study has highlighted their perceptions on how socio-political, health system and behavioural factors impact on their and individual patients’ ability to manage cardiovascular risk factors in a setting affected by protracted conflict. Based on the data presented, this discussion will bring to the fore the complex ways in which these determinants are entangled and why understanding this entanglement is crucial in order to develop adapted and context-specific health interventions for the field of CVD secondary care.

### Complex entanglements: Socio-political, health system and individual factors

In terms of *political factors*, respondents emphasised first and foremost the detrimental health impact of the Israeli occupation by linking it to high emotional stress levels and low quality of life, economic hardship, loss of free movement and a barrier to the access and provision of healthcare. *Social factors* affecting CVD secondary care prevention were mainly related to daily stressors [65] such as low salaries and high workload as well as gender- and age-related factors. Both political and social determinants were perceived to directly and indirectly influence *behavioural factors*, particularly smoking, unhealthy diet and an increasingly sedentary lifestyle.

The results also made apparent the ways in which healthcare professionals perceive these multiple-level factors to come together in people’s lives and, thus, shape individual health outcomes as well as treatment effectiveness. For instance, they explained how smoking, a behavioural risk factor, is linked to (a) the military occupation in that people recur to smoking as a form of stress relief; (b) enjoyment and a way of socialising among peers; and (c) family tradition as younger people follow in the footsteps of their parents and grandparents. Similarly, a sedentary lifestyle was referred to as more than just a behavioural risk; it was connected to gendered norms, cultural habits, lack of adequate spaces for exercise and a restriction of movement especially for those living close to checkpoints or illegal settlements (for comparison see also [66]). These perceptions are mirrored in epidemiological research in the region, which has found strong links between military occupation, economic hardship and psychosocial stress [67], hypertension and obesity [52] and cardiac health [68].

Health providers made apparent that in order to provide adequate treatment, it is crucial for the health system to be responsive to the complex links between societal- and individual-level health factors. Indeed, public health messages focusing primarily on individual behaviour change were not believed to be sufficient in a context where people have a lack of control over their lives due to structural and social barriers. This notion is reinforced by a Palestinian Ministry of Health [61] report stating, “Palestinian society and the PNA [Palestinian National Authority] have little control over the social determinants of health” due to “deep inequities of power and wealth between Palestine and Israel with the latter controlling most aspects of daily life” (p. 9).

Consequently, a broader set of services is required that address, besides medical problems, also social, economic and political factors or, what has been called, the “causes of the causes” of ill health [69, 70]. This is in line with recommendations of the WHO [67] which foreground improving social determinants of health and inequities by tackling daily living conditions; inequitable distribution of power, money and resources; and measuring and understanding the problem and assessing the impact of action. In other words, as the WHO’s Director General stated, “Healthcare is an important determinant of health. Lifestyles are important determinants of health. But (…) it is factors in the social environment that determine access to health services and influence lifestyle choices in the first place” [14]. While we agree with this overall statement, it needs to be understood that, in Palestine, a focus on social determinants of health can only be fruitful, if it is tightly linked to human rights and justice [71]. Indeed, Giacaman and colleagues write:

“Hope for improving the health and quality of life of Palestinians will exist only once people recognize that the structural and political conditions that they endure in the occupied Palestinian territory are the key determinants of population health” (p. 847).

### Proposed solutions for improving CVD secondary care

While study participants were aware of the intricacies outlined above, their proposed solutions for improving CVD outcomes were less concerned with larger structural issues than with behavioural change and medical treatment. The reason for this could be at least twofold. First, it could be due to their professional training considering that cardiologists in secondary care tend to be more likely to see their role as diagnostic and located in the management of acute events. They may, therefore, feel less suited to consider prevention and management of long-term conditions let alone working across sectors to deliver more holistic approaches to healthcare. Second, it could also be related to their own sense of powerlessness in the face of political and structural violence.

What health providers agreed on was that the increase and detrimental consequences of CVD should be addressed by the Ministry of Health at a systems and policy level. In particular, they focused on the importance of prevention, system development and policy improvement. First, practitioners suggested shifting action from treatment to health promotion by focusing on prevention through awareness-raising and education campaigns to inform the public about adverse risks, related to as smoking, obesity, and lack of physical activity. While such approaches have been shown to prevent CVD, it has to be recognised that they have crucial limitations when implemented in conflict-affected areas. First, checkpoints and the Israeli settlements can make it difficult to be physically active outdoors due to threats, harassment and controls. Second, the environment is often considered inappropriate for physical exercise for adults and children alike. For instance, a study by Abdul-Rahim and colleagues [72] has pointed to a lack of sex-segregated facilities that prevents particularly women from participating in sports. Interestingly, however, studies have also shown that living in rural areas in Palestine can be a protective factor due to the fact that agricultural labour is more physically demanding and there is better access to healthy foods [41]. At the same time, it has to be acknowledged that farming communities (particularly those located in Area C of the West Bank) are regularly affected by land confiscations and the control of water sources by Israel – a situation which has been linked to a change in diet generally from one that basically relied on locally grown food including fruit and vegetables to one consisting of processed, fatty, sugary, fast food [52].

Second, practitioners called for a significant improvement of primary care services in order to alleviate the pressure on secondary care. According to them, primary care services were wholly inadequate in terms of risk factor monitoring and follow-up, leading to patients choosing to present directly to secondary care services. To solve this dilemma, the majority of participants highlighted the need for a more cohesive interface between primary and secondary care provision by improving communication between these two services, and between specialists involved in a patient’s care. Besides improved communication, they also demanded more streamlined referral processes, the implementation of multi-disciplinary teams, a greater number of specialists, and additional resources for staffing. While these demands are certainly well placed, they have to be viewed within a context of resource scarcity (excluding salaries, the health budget amounts to about $322,729,780) where the MoH is confronted with a yearly budget deficit of around 45%. Most of the available budget is already spent on medical referrals, medication and medical and laboratory supplies which leaves little room for large-scale system development, let alone addressing upstream determinants of health [60].

Third, policy development was considered an important measure to improve NCD prevention and treatment. Recently, the Ministry of Health has indeed formulated a vision to develop a multi-sectoral strategy for NCD prevention and control in the region [53]. According to the document, such a strategy would address tobacco control and tobacco taxes, health nutrition (including regulation marketing of food and non alcoholic beverages to children), salt intake, saturated fat intake, and physical activity. Yet, it remains unclear how exactly these measures will be implemented and what the challenges may be. For instance, any policy around tobacco control may be difficult to realise considering that the oPt is not yet a signatory to the Framework Convention on Tobacco Control (FCTC) as it was not deemed to be a State when the Convention was signed (2003–4). Nevertheless, the oPt has adopted an anti-smoking law, raised the taxes on tobacco products, banned the public promotion of tobacco and placed written health warnings on products while trying to combat tobacco smuggling despite the fact that it is unable to completely control the border crossings [60]. This last point highlights once again that the implementation of a multi-sectoral NCD prevention strategy cannot be viewed in isolation, but in a context where the Palestinian government has limited room for manoeuvre due to the Israeli occupation and related political fragility and insecurity.

## Conclusion

Due to globalisation and population ageing, non-communicable diseases such as cardiovascular disease are on the rise worldwide. This trend can also be noticed in conflict-affected areas where, until recently, infectious diseases and injuries were the main drivers of morbidity and mortality. Consequently, medical and social intervention strategies will have to be adapted to reflect these emerging trends while also taking local contextual factors into account, which shape, often in very distinct ways, health needs, help- and health-seeking behaviours and access to healthcare. This is of particular relevance for the oPt which has been under Israeli occupation for over 50 years. This conflict has not only impacted on the ability of health providers to deliver healthcare in an environment fraught with barriers, but also on the Palestinian people to access such healthcare and to be motivated to engage in adopting a healthier lifestyle, the result of which is evident from the increase in CVD across the region.

To provide meaningful healthcare in such a complex context, it is important to generate inter-disciplinary, locally relevant evidence related to CVD prevalence rates, health-seeking behaviour, access to health and social care, and outcomes of intersecting medical and socio-political intervention strategies. Moreover, it is important to generate locally relevant and reliable data on CVD and treatment effectiveness in order to be able to estimate costs, resources required, policy and decision-making management. The absence of such data, in turn, can significantly hamper the effectiveness of primary and secondary care services. Thus, improved pathways need to be established between inter-disciplinary data collection and inter-sectoral healthcare delivery with a view to improve health, equity and justice in the long-term.

## Abbreviations

CVD: Cardiovascular disease
ER: Emergency room
ICA: Israeli civil administration
LMIC: Low- and middle-income country
MENA: Middle East and North Africa
MoH: Ministry of health
NCD: Non-communicable disease
NGO: Non-governmental organisation
oPt: Occupied Palestinian territory
PNA: Palestinian national authority
PNSHP: Palestinian national strategic health plan
UK: United Kingdom
WHO: World Health Organisation
